# A Case of Fibrillary Glomerulonephritis

**DOI:** 10.7759/cureus.28250

**Published:** 2022-08-22

**Authors:** Maamannan Venkataraj, Phani P Morisetti

**Affiliations:** 1 Internal Medicine, Louisiana State University Health Sciences Center, Shreveport, USA; 2 Internal Medicine/Nephrology, Louisiana State University Health Sciences Center, Shreveport, USA

**Keywords:** nephrology, fibrillary glomerulonephritis, fibrillary, myeloma, gloerulopnephritis

## Abstract

Fibrillary glomerulonephritis (FGN) is a very rare manifestation of glomerulonephritis characterized by the presence of deposits of randomly oriented microfibrils (10-30 nm size) in the glomeruli and visible on electron microscopy. Our patient is a 63-year-old African American male who presented with a past medical history of cirrhosis; he was initially suspected to have hepatorenal syndrome, but on kidney biopsy, and was diagnosed with FGN. Possible multiple myeloma was suspected due to its strong association with FGN and an elevated serum kappa-lambda ratio in the patient. This was confirmed by bone biopsy to be smoldering myeloma.

## Introduction

Fibrillary glomerulonephritis (FGN), first identified in 1977, is a very rare manifestation of glomerulonephritis that occurs in only 1% of all glomerulonephritis kidney biopsy specimens [1]. It typically manifests after the sixth decade and is characterized by the presence of deposits of randomly oriented microfibrils (10-30 nm size) in the glomeruli, visible with electron microscopy of the biopsy specimen [2]. Unlike in amyloidosis, these fibrils are typically congo red-negative and common histochemical dyes do not adequately stain them. As an immune-mediated glomerulonephritis, the fibrils in FGN also stain intensely for IgG and C3 [3]. Fibrillary, glomerulonephritis is identified more often in Caucasians compared to African Americans, with a ratio of 8.3:1 [4]. It also occurs in association with hepatitis C and multiple myeloma and usually has a very poor prognosis [3].

Our patient is unique in that he is a 63-year-old African American male initially suspected to have hepatorenal syndrome due to concomitant cirrhosis. He was eventually identified as having FGN based on electron microscopy findings in the renal biopsy.

## Case presentation

Presenting complaint

A 63-year-old African American male presented with a past medical history of cirrhosis, hepatitis C with an undetectable viral load, positive antinuclear antibody (ANA) and Sjögren's syndrome type A (SSA), hypertension, hyperlipidemia, iron-deficiency anemia, and *Helicobacter pylori* infection. He presented to the ED after a GI clinic appointment, as bloodwork revealed elevated creatinine of 3.3 mg/dL (baseline=1.9). The patient was advised to come to the emergency department due to a suspicion of acute kidney injury and hepatorenal syndrome.

In the emergency department, the patient complained of abdominal distension and pain with worsening lower limb edema. He also reported episodes of nausea and vomiting over the last several days. The patient stated that he was still producing urine, but urine output was slightly decreased from his baseline. He denied any difficulties with urination, or abdominal pain, fever, or chills. His vital signs were stable. 

He was oriented to person, place, and time and was not in distress. The abdominal examination showed a distended abdomen with normal bowel sounds and no mass, tenderness, rebound, or guarding. Pitting pedal edema of both lower extremities was seen. All other systems' examinations were normal.

Treatment plan

After admission, an initial therapeutic large-volume paracentesis was done on January 3, 2020, with 8.5 L of fluid removed. Fractional excretion of urea was 5.5% and the fractional excretion of sodium was less than 1%, indicating a prerenal picture. Due to the existence of a prerenal cause of elevated creatinine and pre-existing cirrhosis, there was a high suspicion of hepatorenal syndrome for which albumin challenge was attempted.

Intravenous albumin of 1 g/kg/day was given for two days to evaluate improvements in creatinine and to diagnose hepatorenal syndrome. The albumin challenge did not have significant improvement, indicating an increased likelihood of hepatorenal syndrome. On the advice of nephrology, repeat urine sodium and urine protein-creatinine ratios were ordered, which were 19 mEq/day and 2.3 gm/day, respectively, indicating sub-nephrotic proteinuria. However, this proteinuria greatly exceeded the guideline given by the International Ascites Club for hepatorenal syndrome (500 mg/day) [5]. An albumin-creatinine ratio of 0.489 mg/mg as compared to the much-higher urine protein-creatinine ratio indicated a high likelihood of the existence of para-proteinuria. Urine microscopy merely showed many hyaline casts, but was otherwise normal.

On the advice of rheumatology, an in-depth autoimmune panel (IFA) was performed, the results of which were that the patient tested positive for anti-Smith antibodies and negative for anti-Sjögren's syndrome type B (SSB), ds-DNA antibody (Ab), anti-centromere Ab, SCL-70 Ab, ribonucleoprotein (RNP) Ab, anti-smooth muscle Ab, and ANA IgG.

Another therapeutic large-volume paracentesis was performed on January 10, 2020, with 7.2 L removed. Iron deficiency was corrected with four doses of 125 mg of intravenous iron dextran. The *H. pylori* regimen was completed. An abdominal MRI was performed and merely reconfirmed the existence of cirrhotic and hepatocellular changes in the liver with no other positive findings.

*Kidney Biopsy* 

This was subsequently performed on January 14, 2020, to isolate the cause of proteinuria and it showed fibrillary glomerulonephritis with focal mild interstitial fibrosis. All glomeruli show advanced deposition of electron-dense thick fibrils with rare open loops.

Microscopic examination

**Figure 1:**
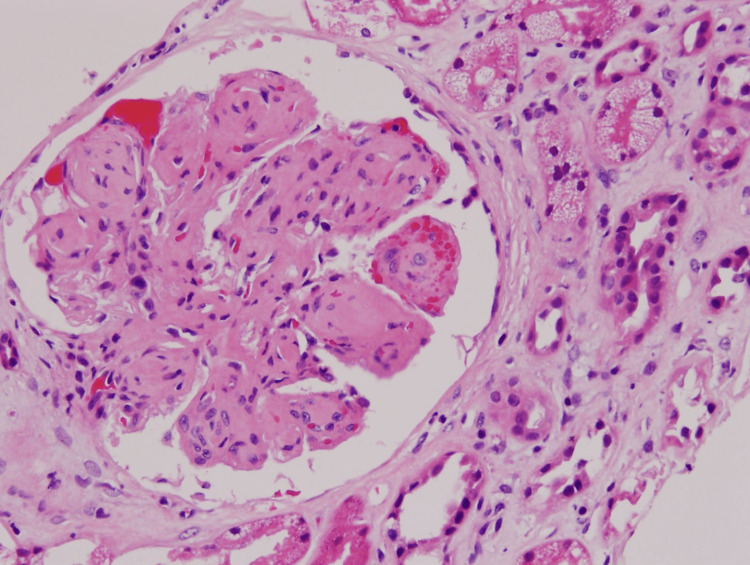
Glomeruli show advanced mesangial expansion.

**Figure 2:**
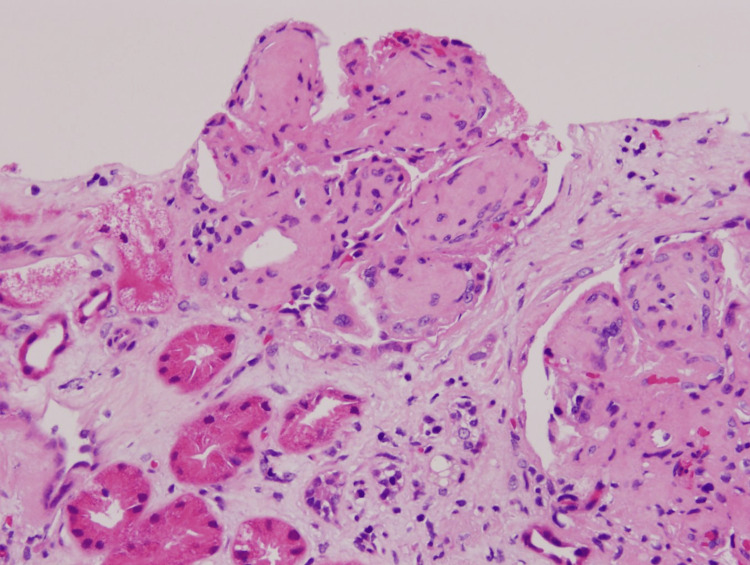
Glomeruli show lobulation of tufts and nodular change.

**Figure 3:**
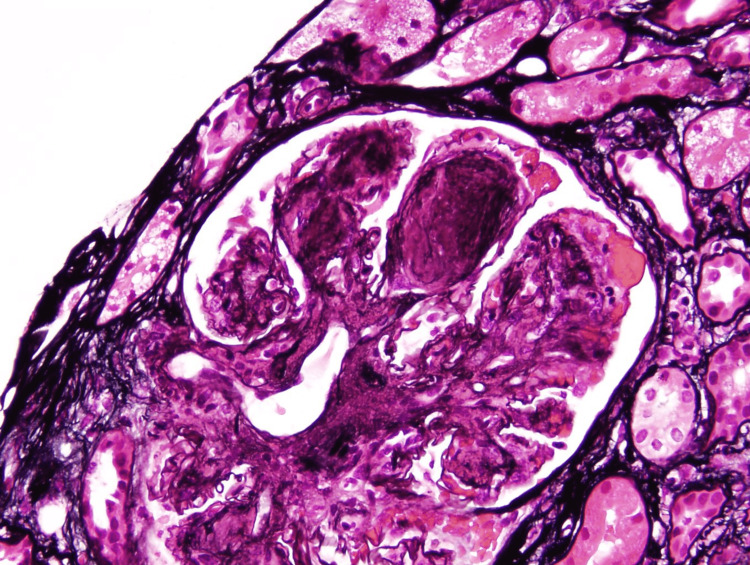
Silver stain - glomeruli show mesangial proliferation.

**Figure 4:**
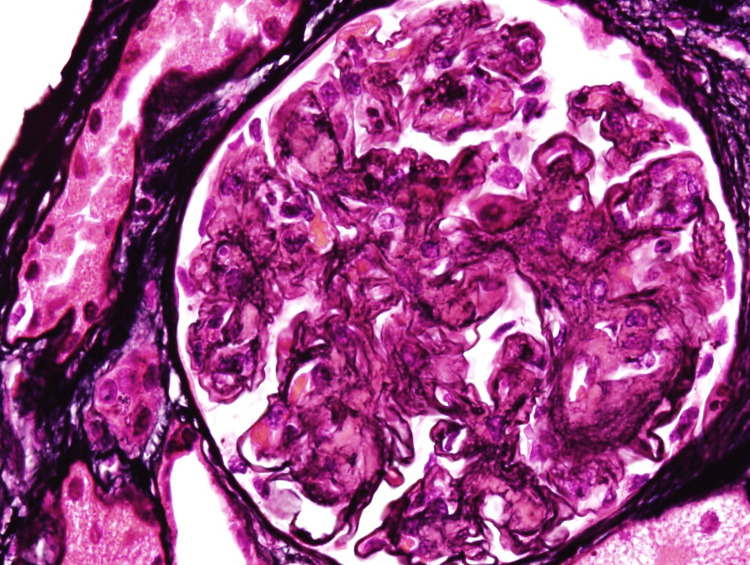
Silver stain - glomeruli show thickening and segmental duplication of capillary membranes.

Multiple sections and special stains (periodic acid-Schiff {PAS}, trichrome, and silver) revealed renal cortex. Twelve glomeruli were present. All glomeruli show advanced mesangial expansion from sclerotic nodules and rare open loops (Figures 1, 2). In the open loops, thickening and segmental duplication of capillary membranes are seen on silver stained sections (Figures 3, 4). Some loops contain fragmented red blood cells. No crescents, proliferation of capillary cells, or necrosis of capillary tufts are identified and no neutrophils or microthrombi are present. Focal mild interstitial fibrosis is present on trichrome-stained sections. There is no significant inflammatory infiltration in the interstitium. The arterioles reveal mild fibrointimal thickening. Congo red and thioflavin-T stains are negative in the glomeruli, interstitium, and vasculatures. 

Immunofluorescence

**Figure 5:**
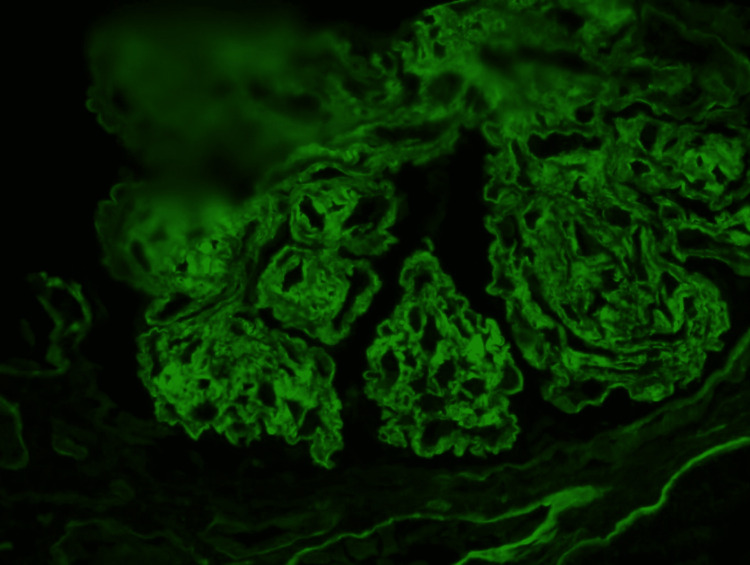
Glomeruli show positive reaction to IgG.

**Figure 6:**
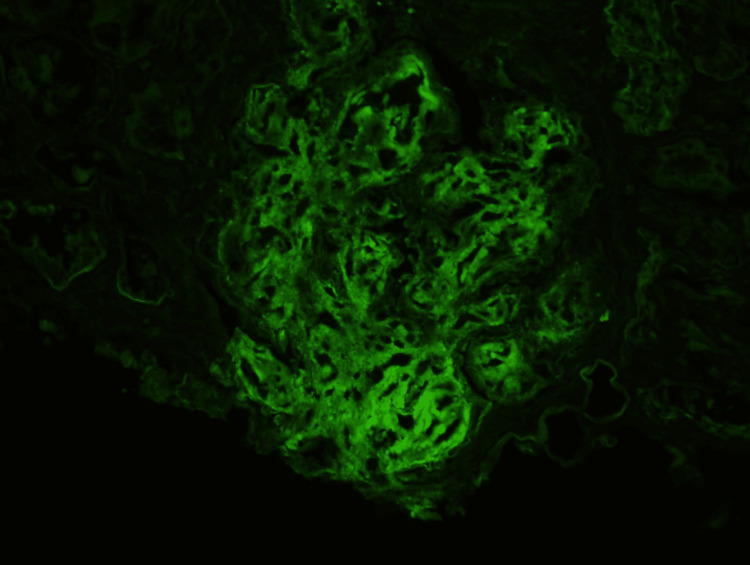
Glomeruli show positive reaction to C3.

The frozen tissue contains several glomeruli on albumin-stained sections. There is a diffuse 2 to 3+ reaction for IgG in the glomeruli (Figure 5). There is also segmental-to-diffuse 2+ reaction in the glomeruli for C3 (Figure 6). There is no reaction for IgM, IgA, C1q, kappa, lambda, and fibrin. Protein casts are positive for kappa and lambda with equal intensity. These findings indicate IgG and C3 deposition in the glomeruli. 

Electron microscopy

**Figure 7:**
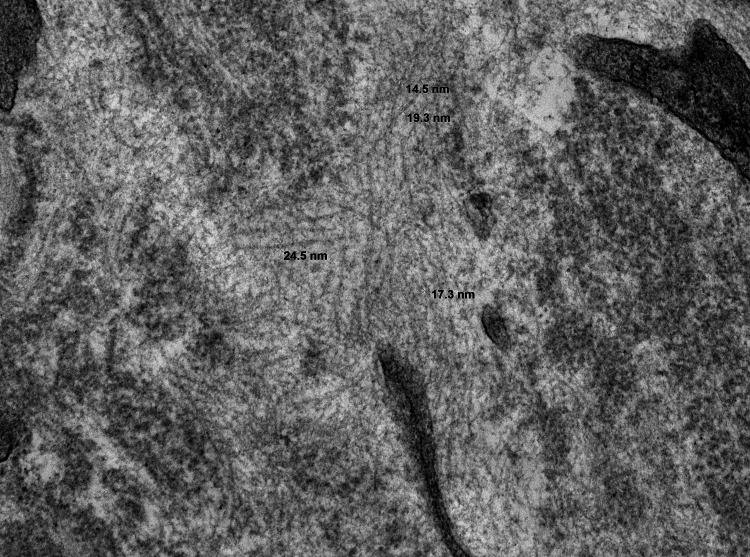
EM reveals diffuse accumulation of randomly oriented fibrils of varying sizes. EM: electron microscopy

**Figure 8:**
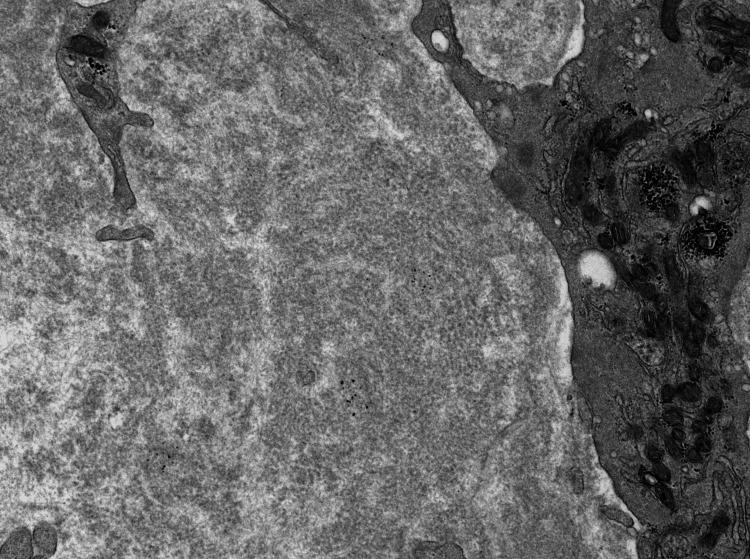
EM reveals diffuse accumulation of thick fibrils in the glomeruli.

The one-micron sections contain two glomeruli and ultrastructural preservation is excellent. Ultrastructural examination shows diffuse effacement of podocyte foot processes. The capillary loop basement membranes and mesangial matrix are substantially altered by the accumulation of coarse fibrillary deposits (Figures 7, 8). The fibrillary deposits are much thicker than amyloid deposits (18-25 nm). These findings indicate the presence of fibrillary deposits within mesangial and capillary loop areas.

After identifying fibrillary glomerulonephritis as the cause of this patient’s elevated creatinine, we decided to rule out possible secondary causes of FGN. Hepatitis C viral load and free light chain assay showed an undetectable viral load and an abnormally increased kappa-lambda light chain ratio of 824. Hematology was consulted regarding the elevated kappa-lambda light chain ratio. Serum protein electrophoresis and serum immunotyping revealed a monoclonal protein identified as (IgA kappa) and cloning in the beta 1 region. Urine immunotyping reconfirmed the elevated kappa light chains as present in urine.

X-ray bone scan was negative for osteolytic lesions, and nuclear medicine bone scan showed only mild evidence of bone disease. However, due to the elevated kappa light chains in the serum, as well as the high association of multiple myeloma with FGN, a bone biopsy was performed.

There was a mild worsening of abdominal distension with an increase in serum creatinine, aspartate transaminase (AST), and alanine aminotransferase (ALT). Paracentesis with cytology was completed, leading to improved creatinine, AST and ALT. As the patient was asymptomatic, we subsequently discharged him with instructions regarding follow-up.

Final pathologic diagnosis

Bone Biopsy Report

The bone biopsy report indicated a plasma cell myeloma (70% cellularity) with kappa light chain restriction. There was normocellular (40%) bone marrow with reduced trilineage hematopoiesis, absent storage iron without ringed sideroblasts, and severe microcytic anemia with rouleaux formation.

Based on recommendations of the 2017 WHO classifications, the findings are most consistent with active or symptomatic myeloma if (1) serum paraprotein is present and (2) there is evidence of myeloma-related organ or tissue impairment or symptoms. The findings are most consistent with smoldering or asymptomatic myeloma if there is no evidence of myeloma-related organ or tissue impairment or symptoms.

Despite evidence of serum paraproteinemia, there was no evidence of myeloma-related impairment of organs and tissues. Hence, we submit that our patient has a case of smoldering myeloma.

## Discussion

Fibrillary glomerulonephritis, known as such due to its characteristic ultrastructural feature of randomly arranged fibrils (approx. 20 nm in diameter) is a pathologically unique form of glomerular disease. These fibrils are composed of a complex of antibodies and antigens, as suggested by their intense staining for IgG and C3 [6].

It was first described as a glomerulopathy separate from amyloid in 1977 by Rosenmann and Eliakim. Unlike amyloidosis, this glomerulonephritis was characterized by larger fibrils (20 nm diameter) that did not stain with thioflavin or Congo red [1]. It was also differentiated ultrastructurally from immunotactoid glomerulopathy at a later date by the random orientation of the fibrils, as opposed to microtubular appearance of fibrils with a hollow core of immunotactoid glomerulopathy [7].

Proteinuria is common in almost all patients with this disease, with a mean urinary protein ranging from 4.1 to 7.3 g/day. Full nephrotic syndrome is seen in 25-69% of patients and is the most common manifestation. Microhematuria is also seen in 52-95% of patients. Renal insufficiency at diagnosis is seen in around 33% of patients, with mean serum creatinine ranging from 2.1 to 3.7 mg/dL [3].

Most patients also have distinctive IgG4-dominant immune deposits visible via immunofluorescence microscopy. Despite the presence of IgG4-staining fibrils, the concentrations of various serum IgG subclasses, including IgG4, are usually within normal limits. Hypergammaglobulinemia is typically absent. However, monoclonal gammopathy is seen in 4-16% of patients. Serum cryoglobulin, rheumatoid factor, and hypocomplementemia are typically not present [8].

Pathologic characteristics

Light Microscopy

Mesangial glomerulonephritis is the most commonly seen pattern of glomerular injury. Mesangial expansion is characteristic of this subtype and is caused by mesangial deposits and sclerosis, as well as various degrees of mesangial hypercellularity (Figure 1) [9]. Membranoproliferative glomerulonephritis (MPGN) is the second most commonly seen pattern, characterized by glomerular basement membrane cellular interposition double contouring at a segmental or global level [3]. This pattern is also associated with mesangial sclerosis, deposits, and hypercellularity, and, thus could possibly represent a stage of advanced disease. The documented conversion from mesangial GN to MPGN over a span of time supports this hypothesis [10]. Our patient shows advanced mesangial expansion in all visible glomeruli (indicative of mesangial GN). This pattern is associated with one of the best long-term prognoses [6].

Immunofluorescence Microscopy

Intense positivity for polyclonal IgG and C3 is seen in the mesangial and glomerular capillary walls in the majority of cases. Our patient is a typical example of such. Glomerular staining for IgA, IgM, and C1q is rarely seen and, when present, is less intense.

The texture of glomerular deposits is typically smudgy, although pseudolinear staining of the glomerular basement membrane, mimicking anti-glomerular basement membrane nephritis, is seen in a minority of cases [11]. However, dominant staining for IgG4 is seen on IgG subclass analysis in most cases of fibrillary glomerulonephritis [12].

Fibrillary glomerulonephritis, initially identified as idiopathic, has been revealed to have frequent association with malignant neoplasm, hepatitis, and autoimmune disease. Up to a quarter of patients with FGN have been diagnosed with malignant neoplasm, more commonly solid than hematologic [13]. The malignant neoplasm may herald, occur in parallel with, or trail the diagnosis of FGN. In AL amyloidosis, monoclonal light chains interact with other proteins to form a β-pleated sheet in the glomeruli and tubules, causing 7- to 10-nm amyloid fibrils to be visible on electron microscopy [14]. Possibly a similar mechanism is at work here in fibrillary glomerulonephritis. However, the exact nature of the association between fibrillary glomerulonephritis and multiple myeloma is still not completely elucidated.

Fibrillary glomerulonephritis has been found to have an association with hepatitis C viral infection, and this association has been found to be stronger in people of African descent. Due to this strong potential association, HCV viral load tests were repeated for our patient, which were found to be negligible. It has been hypothesized that immune system stimulation by chronic HCV infection could drive the progression of FGN. Unlike membranoproliferative glomerulonephritis (MPGN) which is also associated with HCV, FGN does not typically present with hypocomplementemia or cryoglobulinemia [15].

Fibrillary glomerulonephritis does not currently have any curative therapy. Owing to its efficacy in slowing the disease progression in other glomerulopathies, renin-angiotensin system blockade has been advised in all patients with fibrillary glomerulonephritis. In fact, a minority of patients have been shown to undergo remission purely on renin-angiotensin blockade alone [16]. However, due to the potential autoimmune nature of fibrillary glomerulonephritis, immunosuppressive therapy is usually added to most drug regimens. Steroids, cyclosporine, cyclophosphamide, and mycophenolate mofetil are only a few of the many immunosuppressive medications that have been given to patients with FGN. However, in the majority of patients, immunosuppression does not seem to slow prognosis of the disease. Further randomized controlled trials must be conducted to assess the efficacy of immunosuppressive agents in FGN to arrive at a clearer picture of their usage. Renal prognosis is poor in FGN. Within four years of diagnosis, almost half progress to end-stage renal disease (ESRD). Various predictors of progression are as follows: advanced age, markedly elevated serum creatinine, the presence of proteinuria at diagnosis, the glomerular pattern (e.g., MPGN vs. mesangial GN), and the presence of extensive glomerulosclerosis, and interstitial fibrosis. FGN in African American patients with prior HCV infection (as in our patient) is associated with a poorer prognosis However, even in the absence of immunosuppressive therapy, remission occurs in only a minority of patients with FGN. This is particularly seen in younger patients with normal serum creatinine levels at the time of diagnosis and absence of biopsy evidence of chronic disease [3].

## Conclusions

Underlying autoimmune diseases, malignancy, and hepatitis are associated with fibrillary glomerulonephritis. Prognosis is poor in most cases, with progression to ESRD, but remission may occur in a minority of patients. Age, degree of renal impairment at the time of diagnosis, and degree of glomerular scarring are some predictors of renal survival. A regimen of renin-angiotensin blockade and immunosuppression is commonly administered in FGN; however, it is not particularly effective. Due to the rarity of fibrillary glomerulonephritis, more research needs to be done on the subject.
